# Characterization of the immune system of Ellegaard Göttingen Minipigs - An important large animal model in experimental medicine

**DOI:** 10.3389/fimmu.2022.1003986

**Published:** 2022-09-20

**Authors:** Clara P.S. Pernold, Emil Lagumdzic, Maria Stadler, Kerstin H. Mair, Sven Jäckel, Michael W. Schmitt, Andrea Ladinig, Christian Knecht, Sophie Dürlinger, Heinrich Kreutzmann, Vera Martin, Spencer Sawyer, Armin Saalmüller

**Affiliations:** ^1^ Institute of Immunology, Department of Pathobiology, University of Veterinary Medicine Vienna, Vienna, Austria; ^2^ Christian Doppler (CD) Laboratory for Optimized Prediction of Vaccination Success in Pigs, Institute of Immunology, Department of Pathobiology, University of Veterinary Medicine Vienna, Vienna, Austria; ^3^ Chemical and Preclinical Safety, Merck KGaA, Darmstadt, Germany; ^4^ University Clinic for Swine, Department for Farm Animals and Veterinary Public Health, University of Veterinary Medicine Vienna, Vienna, Austria

**Keywords:** ellegaard göttingen minipigs, immune system, T cell differentiation, memory, postnatal development

## Abstract

Interest in Ellegaard Göttingen Minipigs (EGMs) as a model in experimental medicine is continuously growing. The aim of this project is to increase the knowledge of the immune system of EGMs as information is still scarce. Therefore, we studied the postnatal maturation of their immune system from birth until 126 weeks of age. For the first 26 weeks of the study, animals were kept under pathogen-reduced conditions (SPF) and afterwards under conventional housing conditions. The development of the immune system was analyzed by monitoring changes in total numbers of leukocytes and lymphocytes of ten individuals and the composition of leukocyte populations by multi-color flow cytometry (FCM). We followed the presence of monocytes using monoclonal antibodies (mAbs) against CD172a^+^ and CD163^+^ and B cells based on the expression of CD79a. NK cells were distinguished as CD3^-^CD16^+^CD8α^+/dim^ cells and further subdivided using NKp46 (CD335) expression into NKp46^-^, NKp46^+^, and NKp46^high^ NK cells. T-cell receptor (TCR) γδ T cells were defined by the expression of TCR-γδ and different subsets were determined by their CD2 and perforin expression. TCR-αβ T cells were classified by their CD8β^+^ or CD4 expression. For monitoring their differentiation, expression of CD27 and perforin was investigated for CD8β^++^ T cells and CD8α together with CD27 for CD4^+^ T cells. We clearly detected a postnatal development of immune cell composition and identified phenotypes indicative of differentiation within the respective leukocyte subsets. Examination of the development of the antigen-specific immune system after transfer to different distinct housing conditions and after vaccination against common porcine pathogens such as porcine circovirus 2 (PCV2) revealed a markedly increased presence of more differentiated CD8^+^ and CD4^+^ T cells with central and effector memory T-cell phenotypes. To complement the findings, a PCV2 vaccine-specific antigen was used for *in vitro* restimulation experiments. We demonstrated antigen-specific proliferation of CD4^+^CD8α^+^CD27^+^ central and CD4^+^CD8α^+^CD27^-^ effector memory T cells as well as antigen-specific production of TNF-α and IFN-γ. This study of postnatal immune development defines basic cellular immune parameters of EGMs and represents an important milestone for the use of EGMs for immunological questions in experimental medicine.

## Introduction

The importance of swine and especially Ellegaard Göttingen Minipigs (EGMs) as a model in translational research and experimental medicine is steadily growing (1–9). In particular, they are increasingly recognized as valuable models for toxicity studies and xenograft research (10–13). Initially, EGMs were introduced as a new breed for skin research (12) as their skin closely resembles that of humans. Likewise, EGMs offer similarities to humans in their cardiac, renal, and digestive systems (14) making them a valuable model for human diseases. They are emerging as a promising alternative to non-human primates (NHP) due to lower acquisition and maintenance costs, shorter generation times, and fewer ethical restrictions, although the last point was heavily discussed in "The Rethink Project" by Forster et al. (1, 15). A huge advantage over rodents is that long-term studies can be conducted, which is essential for many questions in experimental medicine and study of disease courses. Further, genetically modified EGMs with adaptations that resemble genetic alterations responsible for human diseases have been generated (16, 17). All this makes EGMs an interesting model, however, knowledge about their immune system is still scarce. As detailed knowledge about the immune system represents an important factor in translational medicine, we studied the postnatal development of the immune system of ten EGMs (five males and five females) from birth to 126 weeks of age. This long-term study allowed us to gain insight into both the postnatal development of the immune system of young piglets, and the long-term development of the immune system over time following immune stimulation after vaccination. Based on the results of a previous study of our research group (18) showing clear phenotypic maturation of porcine NK and T cell subsets in the first six months of life of domestic swine, we aimed to expand the research questions for this EGM study. (I) In addition to NK and T cells, we followed the development of monocytes and B cells over time. (II) We investigated how the immune system develops under low pathogen conditions and without major challenges in the first six months of life, as this will be of great interest for future studies of the immune system for animals housed under SPF conditions or the evaluation of immune modulatory drugs. (III) Further, the effects on the different components of the immune system after transfer to distinct housing conditions and challenges through vaccinations were traced. (IV) To study an antigen-specific immune response in a more experienced immune system after vaccination, T-cell subsets responsible for an antigen-specific immune response and T-cell memory were investigated using Porcine Circovirus 2 (PCV2) ORF2 antigen for *in vitro* restimulation studies of PCV2 vaccinated animals. The main objective of our study was to define reference values and to increase the knowledge about the development of the immune system of EGMs. We determined immunological characteristics of EGMs and discussed possible differences to other species, in order to strengthen the usability of EGMs in preclinical studies.

## Material and methods

### Animals

Ten EGMs (five males and five females) were used in this study. This breed was established in the 1960s in Göttingen, Germany. The background of these pigs are Minnesota Minipigs from Hormel Institute, Austin, TX, USA and Vietnamese potbellied pigs from Wilhelma Zoo, Stuttgart, Germany. In 1965 Vietnamese potbellied pigs from Friedrichsfelde Zoo, East Berlin, Germany were crossed in. Between 1965 and 1969, the German Landrace was crossed in, and the final breed was established (19). Pigs remained at the Ellegaard Göttingen Minipigs A/S breeding facility, Dalmose, Denmark until 29 weeks of age. After that, they were transferred to the University of Veterinary Medicine Vienna, Austria (Vetmeduni). After their arrival in Vienna, male pigs were castrated and all animals were vaccinated against Mycoplasma hyopneumoniae (Ingelvac MycoFLEX®, Boehringer Ingelheim Vetmedica GmbH, Ingelheim, Germany), PCV2 (Ingelvac CircoFLEX®, Boehringer Ingelheim Vetmedica GmbH), and Actinobacillus pleuropneumoniae (APP) (COGLAPIX®, CEVA Tiergesundheit GmbH, Dessau, Germany). All pigs received booster vaccinations after four weeks and were housed in BSL-2 isolation wards separated by sex until five weeks after arrival. Thereafter, animals were kept together under conventional housing conditions. At week 71, an infection with Glaesserella (G.) parasuis occurred and one male animal had to be euthanized for animal welfare reasons. To ensure the health of the herd, pigs received one dose of Draxxin® (Zoetis, Parsippany, USA) and a stock-specific vaccine against G. parasuis (serotype 4, BS-Immun GmbH, Vienna, Austria) (20) twice within one month. In addition, all pigs were boosted with the above-mentioned vaccines ten months after the first vaccinations. The animal study was approved in Denmark by the Danish authorities (license 2019-15-0201-01622) and in Austria by the institutional ethics committee, the Advisory Committee for Animal Experiments (§12 Animal Experiments Act - TVG), and Austrian Federal Ministry of Education, of Science and Research (reference BMBWF-68.205/0198-V/3b/2019).

### Sample collection

During the first six months of the study, blood was collected at two-week intervals starting 2 – 4 days after birth. After transfer to Vienna, pigs were bled starting at week 44 in intervals between two to ten weeks. For blood sampling, animals were fixed with a V-trough or sling (Denmark), or a nose snare (Austria). Blood was collected by puncture of the Vena jugularis externa or the Vena cava cranialis. In Denmark, a BD Vacutainer™ system (Na-Heparin) was used while in Austria blood was collected in heparin tubes (Primavette® V Li-Heparin 10 mL; Kabe Labortechnik GmbH, Nümbrecht-Elsenroth, Germany).

### Isolation of PBMCs

Isolation of peripheral blood mononuclear cells (PBMCs) was carried out using lymphocyte separation medium (LSM, Pancoll human, density 1.075 g/mL (weeks 0-6) or 1.077 g/mL (weeks 8 – 126), PAN-Biotech, Aidenbach, Germany). Centrifugation was carried out for 30 min at 920 × g at room temperature (RT). The isolation protocol was previously described (18, 21, 22).

Whole blood for determination of leukocytes and lymphocytes and isolated PBMCs were counted using Türk`s solution (Merck KGaA, Darmstadt, Germany) and a Neubauer counting chamber (LO - Laboroptik Ltd, Lancing, UK) by microscopy (Nikon ECLIPSE TS100 microscope, Nikon Corporation, Tokyo, Japan) for weeks 0, 2 and 6. For the first week of the study cell counts of only three animals were available. Therefore, a mean of the three samples was used to calculate the total cell numbers for all animals. For weeks 2 and 6, blood of all animals was counted and the percentage of lymphocytes within total PBMCs was determined by flow cytometry (FCM). For all other study days, samples of all animals were counted on a Sysmex XP300 (Sysmex Austria GmbH, Vienna, Austria). Isolated PBMCs were stored at -150°C in a freezing medium containing 50% (v/v) RPMI 1640 with stable glutamine (PAN-Biotech), 100 IU/mL penicillin and 0.1 mg/mL streptomycin (PAN-Biotech), 40% (v/v) fetal calf serum (FCS, Gibco™, Thermo Fisher Scientific), and 10% (v/v) DMSO (Sigma-Aldrich).

### FCM staining of PBMCs

For FCM staining for phenotyping 1.5 – 2.0 × 106 freshly isolated PBMCs per sample were plated into 96-well round-bottom plates (Greiner Bio-One, Kremsmünster, Austria). Information about the monoclonal antibodies (mAbs) and second-step reagents are presented in **Table 1**. The appropriate working concentrations of mAbs and conjugates were previously determined by titrations. In-house fluorochrome labeling or biotinylation was performed as described before (18). If unlabeled and directly conjugated antibodies with the same isotype were used in combination, a sequential staining was performed. After applying unconjugated primary mAbs and isotype-specific dye-conjugated secondary antibodies, free binding sites were blocked by whole mouse IgG molecules (Jackson ImmunoResearch, Suffolk, UK) before applying the directly labeled primary mAbs. Fixable Viability Dye eFluor780 (Thermo Fisher Scientific; MA, U.S.A.) was used for discrimination of dead cells according to manufacturer's protocol. All incubation steps lasted 20 min, with the exception of labeling of intracellular markers with an incubation time of 30 min. All washing steps were performed at 470 × g, at 4°C for 4 min with 200 µL of the appropriate wash buffer. D-PBS (PAN-Biotech, Aidenbach, Germany) supplemented with 10% (v/v) porcine plasma (in-house preparation) was used as buffer for staining and washing for extracellular markers. D-PBS (PAN-Biotech) without additives was used prior to the viability staining. For fixation and permeabilization, eBioscience™ Foxp3/Transcription Factor Staining Buffer Kit (Invitrogen, MA, U.S.A.) was used according to the manufacturer's protocol for detection of intracellular markers and in addition for all samples at the end of the staining protocol to lyse remaining erythrocytes in fresh samples. An FMO control staining for perforin can be found in **Supplementary Figure 1**.

**Table 1:** Antibodies used for FCM analysis.

Antigen	Clone	Isotype	Fluorochrome	Source of primary antibody	Labeling strategy
**Monocytes**					
CD172a	74-22-15A	mouse IgG2b	PE-Cy7	In-house	Secondary antibody^a^
CD163	2A10/11	mouse IgG1	PE	BioRad	Directly conjugated
**B cells**					
CD3	BB23-8E6-8C8	mouse IgG2a	PerCP-Cy5.5	BD Biosciences	Directly conjugated
CD79a	HM57	mouse IgG1	PE	Thermo Fisher Scientific	Directly conjugated
**NK cells**					
CD16	G7	mouse IgG1	FITC	BioRad	Directly conjugated
CD8α	11/295/33	mouse IgG2a	BV421	In-house	Biotin-streptavidin^b^
CD335 (NKp46)	VIV-KM1	mouse IgG1	Alexa 647	In-house^c^	Directly conjugated
CD3	BB23-8E6-8C8	mouse IgG2a	PerCP-Cy5.5	BD Biosciences	Directly conjugated
**γδ T cells**					
TCR-γδ	PPT16	mouse IgG2b	BV421	In-house	Secondary antibody^d^
CD2	MSA4	mouse IgG2a	Alexa488	In-house^e^	Directly conjugated
Perforin	Delta-G9	mouse IgG2b	PerCP-eFluor710	Thermo Fisher Scientific	Directly conjugated
**Cytolytic T cells**					
CD3	BB23-8E6-8C8	mouse IgG2a	PE-Cy7	BD Biosciences	Directly conjugated
CD8β	PPT23	mouse IgG1	PE	In-house	Biotin-streptavidin^f^
CD27	b30c7	mouse IgG1	Alexa647	In-house^c^	Directly conjugated
Perforin	Delta-G9	mouse IgG2b	PerCP-eFluor710	Thermo Fisher Scientific	Directly conjugated
**T helper cells**					
CD4	74-12-4	mouse IgG2b	PerCP-Cy5.5	BD Biosciences	Directly conjugated
CD27	b30c7	mouse IgG1	BV421	In-house	Biotin-streptavidin^b^
CD8α	11/295/33	mouse IgG2a	Alexa 488	In-house	Secondary antibody^g^
CD25	3B2	mouse IgG1	Alexa 647	In-house^c^	Directly conjugated
**Proliferation assays**					
CD3	BB23-8E6-8C8	mouse IgG2a	PE-Cy7	BD Biosciences	Directly conjugated
CD4	74-12-4	mouse IgG2b	PerCP-Cy5.5	BD Biosciences	Directly conjugated
CD8β	PPT23	mouse IgG1	Alexa 488	In-house^e^	Directly conjugated
TCR-γδ	PPT16	mouse IgG2b	Alexa 647	In-house^c^	Directly conjugated
CD27	b30c7	mouse IgG1	BV650	In-house	Biotin-streptavidin^h^
CD8α	76-2-11	mouse IgG2a	PE	BD Biosciences	Directly conjugated
CellTrace™ Violet dye			Thermo Fisher Scientific		
**Intracellular cytokine staining**					
CD3	BB23-8E6-8C8	mouse IgG2a	PE-Cy7	BD Biosciences	Directly conjugated
CD4	74-12-4	mouse IgG2b	PerCP-Cy5.5	BD Biosciences	Directly conjugated
CD8β	PPT23	mouse IgG1	Alexa488	In-house^e^	Directly conjugated
CD27	b30c7	mouse IgG1	BV650	In-house	Biotin-streptavidin^h^
CD8α	76-2-11	mouse IgG2a	PE	BD Biosciences	Directly conjugated
IFN-γ	P2G10	mouse IgG1	Alexa647	BD Bioscience	Directly conjugated
TNF-α	MAb11	mouse IgG1	BV605	BioLegend	Directly conjugated
**Viability and Blocking**					
Fixable Viability Dye			eFluor780	Thermo Fisher Scientific	
whole mouse IgG			** **	Jackson Immuno Research	

a goat-anti-mouse IgG2b-PeCy7, Southern Biotech.

b Streptavidin-BV421, BioLegend.

c Alexa Fluor™ 647 Antibody Labeling Kit,Thermo Fisher Scientific.

d goat-anti-mouse IgG2b-BV421, Jackson Immuno Research.

e Alexa Fluor™ 488 Antibody Labeling Kit, Thermo Fisher Scientific.

f Streptavidin-PE, Thermo Fisher Scientific.

g goat-anti-mouse IgG2a-AF488, Thermo Fisher Scientific.

h Streptavidin-BV650, BioLegend.

For cultivated PBMCs as described in section 2.5 a staining buffer consisting of D-PBS (PAN-Biotech) with 3% (v/v) FCS (Gibco™) was used. For intracellular cytokine staining (ICS), PBMCs were fixed and permeabilized with BD Cytofix/Cytoperm™ Kit (Becton Dickinson, Franklin Lakes, NJ, U.S.A.) according to manufacturer's protocol.

### Proliferation assay

Following defrosting, PBMCs were resuspended in D-PBS (PAN-Biotech) and filtered through a Falcon Cell Strainer 70 μm Nylon (Corning Inc., New York, U.S.A.) before counting. Cells were adjusted to 2 × 10^7^ cells/mL and 1 mL of a 5 µM CellTrace™ Violet solution (CTV, Thermo Fisher Scientific) was added for each mL of cell suspension, followed by immediate vortexing. The suspension was incubated for 10 min at 37°C in a water bath and briefly vortexed several times. Thereafter, 2 mL FCS (Gibco™) were added to every 2 mL of the cell-dye suspension and cells were incubated for an additional 15 min at RT in the dark. 10 mL of cell culture medium were added and PBMCs were centrifuged as described above. Washing was repeated twice before PBMCs were counted. Validation of the successful cell labeling was performed on a CytoFLEX LX (Beckman Coulter, Brea, CA, U.S.A.). Labeled cells were plated into 96-well round-bottom plates with 2 × 10^5^ cells per well in a total of 200 µL volume. Cells were stimulated with recombinant PCV2-ORF2 protein or GP64 as a baculovirus-expressed control protein (both 4μg/mL, kindly provided by Boehringer Ingelheim Vetmedica GmbH) and cultured for four days (37°C, 5% CO2). Concanavalin A (ConA, 3µg/mL, Amersham plc, Buckinghamshire, UK) stimulated PBMCs and PBMCs cultivated in medium alone served as positive and negative controls, respectively. After four days, microcultures were harvested, cells of the same stimulation group of the same animal were pooled and stained as described above in 96-well round-bottom plates.

### Stimulation of PBMCs for cytokine production

After defrosting, PBMCs were plated in 96-well round-bottom plates with 5 × 10^5^ cells per well in a total volume of 200 µL. Cells were stimulated with a recombinant PCV2-ORF2 protein or GP64 as control protein (both 4 μg/mL, kindly provided by Boehringer Ingelheim Vetmedica GmbH). PMA/Ionomycin treated PBMCs (50 ng/mL and 500 ng/mL, respectively, Sigma-Aldrich, stimulated for four hours) served as positive control and PBMCs cultivated in medium alone served as additional negative control. Cells were cultivated for 18 hours (37°C, 5% CO2). Four hours prior to harvesting, 1 µg/mL Brefeldin A (BD Biosciences) was added to all microcultures to inhibit cytokine release. For the following FCM analyses, at least 12 wells of the same stimulation group were pooled, and cells were washed twice with D-PBS (PAN-Biotech) before staining (described in section 2.4) in 96-well round-bottom plates.

### Data analysis

**Figure 1:**
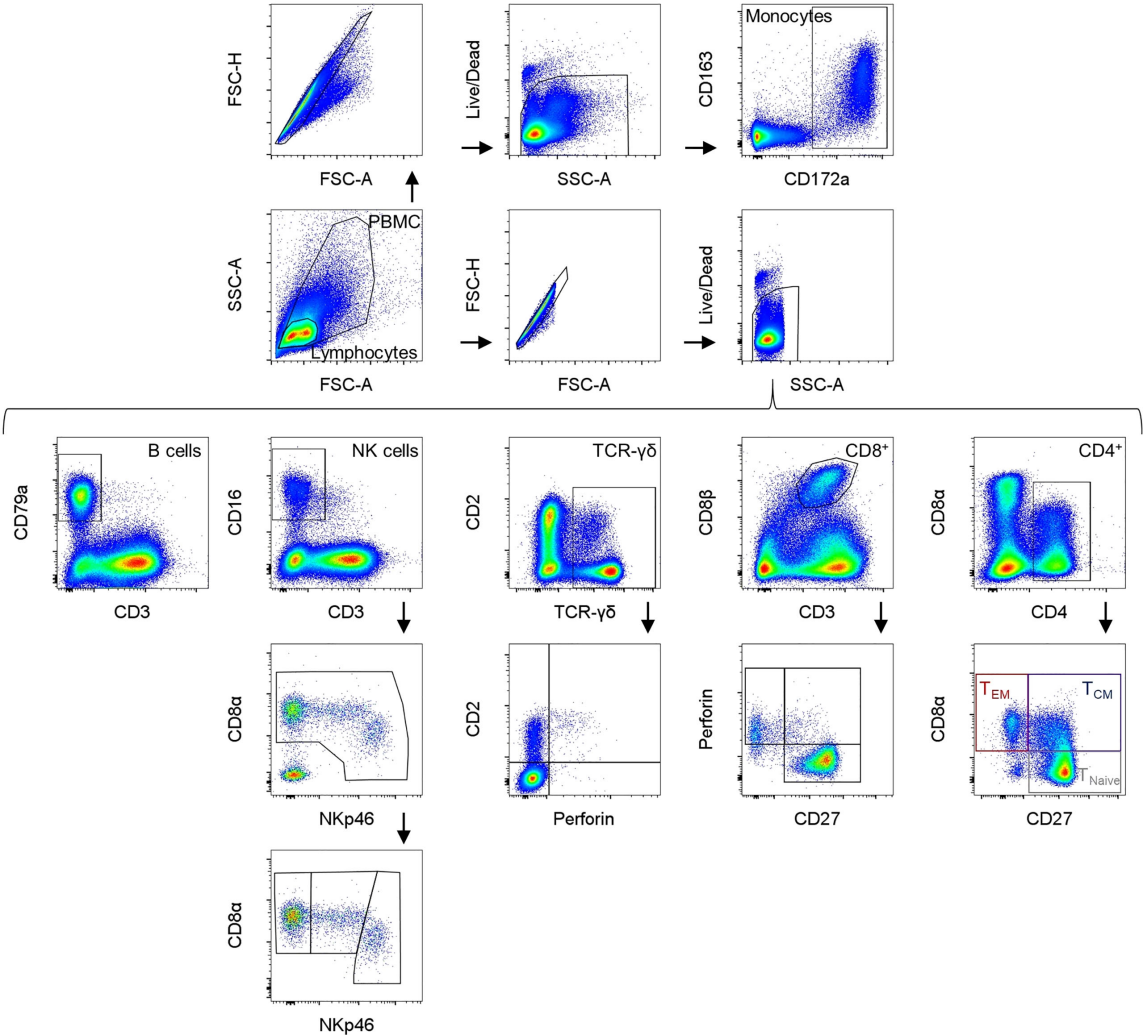
Gating strategies for the different immune cell subsets (a male pig, aged 18 weeks is shown as a representative animal). In the upper row, identification of monocytes is presented using CD172a and CD163 expression. Below, the gating strategy for the different lymphocyte subpopulations is shown. B cells were determined as CD3^-^CD79a^+^. NK cells were characterized as CD3^-^CD8α^+^CD16^+^ and further divided into NKp46^-^, NKp46^+^, and NKp46^high^ NK cells. TCR-γδ^+^ T cells can be divided into three phenotypes: CD2^-^perforin^-^ and CD2^+^ cells that can either be negative or positive for perforin expression (no clear CD2^-^perforin^+^ γδ^+^ T cells were found). Cytolytic T lymphocytes (CTLs) are defined as CD3^+^CD8β^+^ and can be divided into different phenotypes *via* their CD27 and perforin expression. CD4 was used as a pan marker for T helper cells and CD8α and CD27 expression can be used to differentiate between naive CD4^+^CD8α^-^CD27^+^ (T_Naive_) T cells, CD4^+^CD8α^+^CD27^+^ activated and central (T_CM_) and CD4^+^CD8α^+^CD27^-^ effector memory (T_EM_) T cells.

Samples were analyzed using a CytoFLEX LX (Beckman Coulter) equipped with six lasers (U-V-B-Y-R-I). At least 100,000 cells were analyzed per sample. For compensation, single stains were used to set up a compensation library. FCS files were analyzed with FlowJo software version 10.8. (Becton Dickinson). The gating strategy used in all experiments is summarized in **Figure 1**. Mean and median values were calculated using GraphPad Prism version 9 (GraphPad Software Inc., CA, U.S.A.).

## Results

### Identification of cell subsets

To identify the different immune cell subsets, mAbs described in **Table 1** were used. The gating strategy is summarized in **Figure 1**. Monocytes and lymphocytes were gated according to their forward and side scatter parameters (FSC-A/SSC-A), followed by doublet discrimination (FSC-A/FSC-H) and exclusion of dead cells (gating on viability dye negative cells). Monocytes were analyzed by their CD172a^+^CD163^+^ expression (23, 24). B cells were determined as CD3^-^CD79a^+^ lymphocytes (25). NK cells were characterized as CD3^-^CD8α^+^CD16^+^ lymphocytes and further subdivided using NKp46 (CD335) expression into NKp46^-^, NKp46^+^, and NKp46^high^ NK cells (26, 27). TCR-γδ T cells were analyzed by gating on TCR-γδ^+^ lymphocytes that were divided into two major phenotypes: CD2^+^ and CD2^-^ (28–32). To gain more information about potential differentiation and functional activities, these two subsets of TCR-γδ T cells were further analyzed for their perforin expression (33). CD8^+^ T cells were defined as CD3^+^CD8β^++^ lymphocytes, and additionally divided into different subsets according to their CD27 and perforin expression (18, 34–36). CD4 was used as a pan marker to identify the T-helper cell population. For swine, it is known that a substantial portion of CD4^+^ T cells shows a co-expression of CD8α molecules on their surface. Activated and further differentiated CD4^+^ memory T cells keep this CD8α^+^ phenotype and can be further separated by the expression of CD27 into CD8α^+^CD27^+^ central (TCM) and CD8α^+^CD27^-^ effector memory (TEM) CD4^+^ T cells (18, 35, 37–43)

### Total numbers of leukocytes, lymphocytes, monocytes and B cells

**Figure 2:**
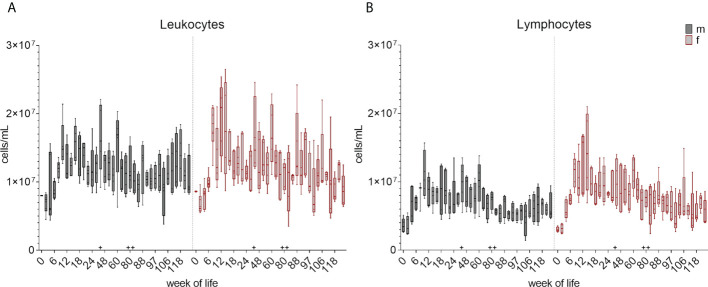
Development of total number of leukocytes and lymphocytes from birth to 126 weeks of age. **(A)** Numbers of leukocytes. Sexes are represented by black (male) and red colors (female). **(B)** Changes of total numbers of lymphocytes. Separation between the sexes was done as for leukocytes. + marks the first bleeding time point after transfer and vaccination, ++ marks the first bleeding time point after booster vaccinations.

**Figure 3:**
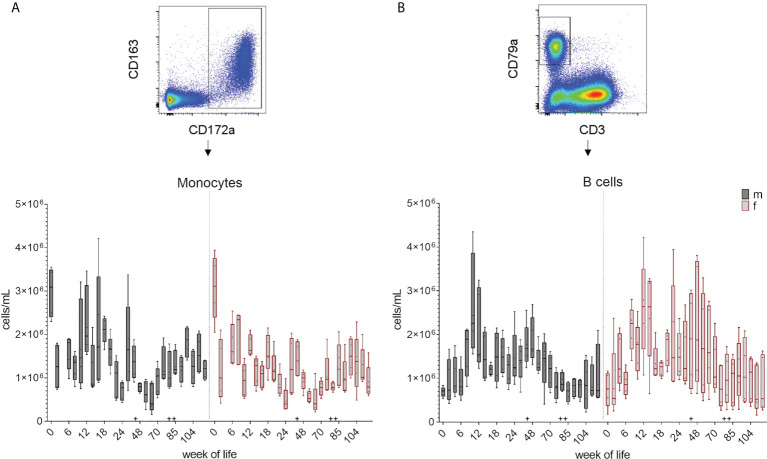
Total number of monocytes and B cells over the study period of 126 weeks. Determination of total cell counts was combined with FCM analyses. A representative EGM (male, 18 weeks of age) was chosen to show the characterization of the subsets. Sexes are represented by black (male) and red colors (female). **(A)** Monocytes were identified using CD172a^+^ co-expressed with CD163^+^. **(B)** Number of B cells were determined by their CD3^-^CD79a^+^ phenotype. + marks the first bleeding time point after transfer and vaccination, ++ marks the first bleeding time point after booster vaccinations.

Leukocyte and lymphocyte counts were determined and calculated at each bleeding time point over 126 weeks as described in section 2.4. The changes in the total cell number during the development of the immune system is summarized in boxplots in **Figure 2** and **Supplementary Figure 2** showing the early postnatal development only. A decrease in the total leukocyte counts between week zero and week two was followed by an increase in median cell counts until week eight in female piglets and week ten in male piglets. An additional increase was observed at week 44. The numbers of lymphocytes showed an increase of median values until week 12 (females) and ten (males), followed by a decrease in the following weeks. An increase in lymphocyte counts was also observed at week 44, and again at week 88 for both leukocytes and lymphocytes. Variation within the pigs was present within the leukocyte counts over the whole study period, but was less prominent for lymphocytes. A similar trend was seen in the total numbers of monocytes (**Figure 3A** and **Supplementary Figure 3A**). A decrease between weeks zero and two, and an increase until week eight (females) and six (males) was observed. Afterwards, a heterogeneous pattern of cell counts was seen until the end of the study for both sexes. Within lymphocytes, B cells were determined to be a prominent subset (**Figure 3B** and **Supplementary Figure 3B**). The total number of B cells decreased for the first time in females in week ten and by week 12 in males, followed by a similar pattern as for monocytes during the study. No age dependent trend was observed over time, except for the end of our study at weeks 118 and 126, where the median values of the total numbers of B cells started to show less variation. Vaccinations and transfer to different housing conditions (as highlighted in section 2.1) resulted in an increased median value of total B cells within both sexes seen in week 44. Following week 48, there was a period of decreasing numbers of measured B cells, which continued for several weeks. Booster vaccinations showed an increase in median values of females.

### Total numbers of NK cells and NKp46/CD8α-defined subsets

**Figure 4:**
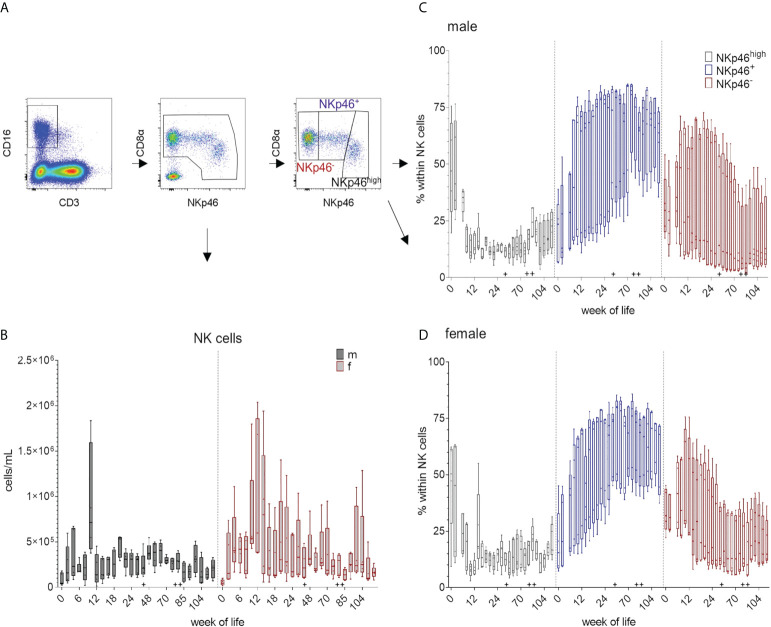
Total number of NK cells and their differentiation over the study period of 126 weeks. Total NK cell numbers were calculated based on the percentages of CD3^-^CD8α^+^CD16^+^ cells in total lymphocytes measured by FCM. **(A)** shows the gating strategy for a representative EGM (male, 18 weeks of age) for the determination of NK cells and the CD8α/NKp46-defined subsets. **(B)** Development of total NK cell numbers over time using CD3^-^CD8α^+^CD16^+^ expression. Sexes are represented by black (male) and red colors (female). Phenotypic analyses of the NK-cell subsets by the expression of CD8α and NKp46 highlighted by different colors. CD8α^dim/+^NKp46^high^ (black), CD8α^+^NKp46^+^ (blue), and CD8α^+^NKp46^-^ (red). Relative changes in the composition of the NK-cell subsets over time are presented for males **(C)** and females **(D)**. + marks the first bleeding time point after transfer and vaccination, ++ marks the first bleeding time point after booster vaccinations.

Total numbers of NK cells increased between weeks zero and four and again between weeks eight and ten. For female pigs, higher variations in total NK cell numbers were observed at most time-points of the study compared to male pigs (**Figure 4B** and **Supplementary Figure 4B**). For EGMs, we could confirm the three NK cell phenotypes previously described for domestic swine (18). The FCM data of one representative animal and the strategy for the analyses are presented in **Figure 1** as well as in **Figure 4A**. Independent of their sexes, all pigs showed higher percentages of CD8α^dim/+^NKp46^high^ cells at the date of birth and following weeks compared to the CD8α^+^NKp46^+^ and CD8α^+^NKp46^-^ phenotypes. While the CD8α^+^NKp46^+^ subset increased during the first weeks of life, the CD8α^+^NKp46^-^ subset decreased. (**Figure 4C, D** and **Supplementary Figure 4C, D**). Towards the end of the study, average median values of CD8α^dim/+^NKp46^high^ frequencies were less than at birth, whereas the median values of the CD8α^+^NKp46^+^ subpopulation had increased. Although a certain diversity, independent of the sex, was present within the animals, the overall tendency demonstrated a related picture over the study period. In all animals an age-dependent decrease of the portion of the CD8α^dim/+^NKp46^high^ subset correlated with an increase of the CD8α^+^NKp46^+^ subset.

### Total number of TCR-γδ T cells and CD2^-^defined subsets

**Figure 5:**
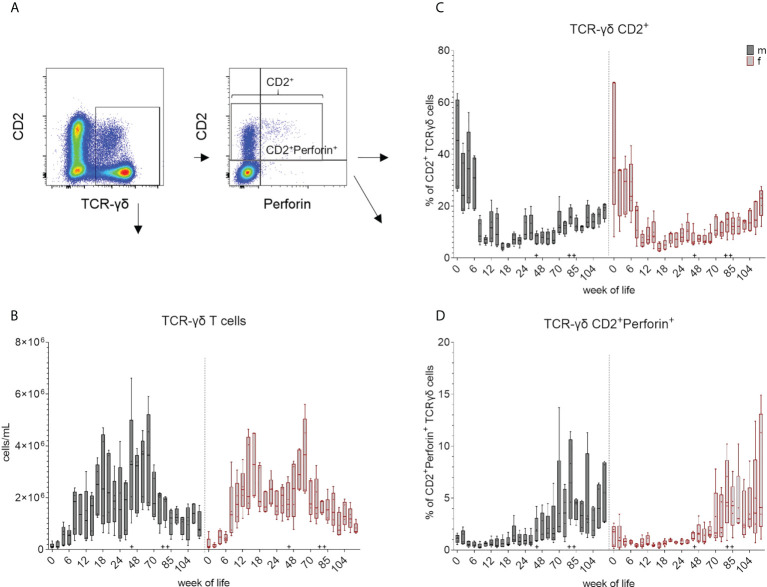
Total number of TCR-γδ T cells and their differentiation over the study period. **(A)** shows the gating strategy for a representative EGM (male, 18 weeks of age) for the analysis of TCR-γδ T cells. Staining of TCR-γδ T cells for CD2 and perforin expression revealed three major subsets: CD2^-^perforin^-^, CD2^+^perforin^-^ and CD2^+^perforin^+^. **(B)** Frequencies obtained in FCM analyses were used for the calculation of the total number of TCR-γδ T cells within the leukocyte population (TCR-γδ T cells/mL) over a study period of 126 weeks. Graphs in **(C)** represent the percentage of CD2^+^ TCR-γδ cells and **(D)** the relative increase of the CD2^+^perforin^+^ TCR-γδ^+^ T cell subset within the total TCR-γδ T cells over a study period of 126 weeks. Sexes are represented by black (male) and red colors (female) in **(B–D)**. + marks the first bleeding time point after transfer and vaccination, ++ marks the first bleeding time point after booster vaccinations.

An increase of median values of total cell number of TCR-γδ T cells was observed during the first weeks of life in all pigs (**Figure 5B** and **Supplementary Figure 5B**). Total counts were comparable between sexes in the first six weeks, although TCR-γδ T cells increased more steadily in females and reached higher median values than in males after three months. Males showed an increase at week 44, followed by a decrease at week 70, while females showed an increase by week 48. Variations between individual animals were lowest at the beginning of the study and decreased again towards the end of the study. As mentioned above, two TCR-γδ subpopulations can be distinguished in domestic swine based on CD2 expression (28, 29, 32, 44). These CD2^-^defined cell subsets could also be identified in EGMs (**Figure 1**). In addition, we confirmed for EGMs that the CD2 expression seems to correlate with the surface expression of the TCR-γδ as previously described for the domestic swine (44). On a single cell level, the CD2^-^ fraction showed a higher expression of their TCRs than the CD2^+^ TCR-γδ T cell subset (**Figure 5A**). Variation of the frequencies of the CD2^+^ subset within the TCR-γδ T cells between animals was higher over the first weeks of life in both sexes and decreased over time showing a trend towards CD2^-^TCR-γδ T cells within the PBMCs. A tendency towards a more continuous increase of CD2^+^ median values did not occur before week 70 and never reached the median values of the beginning (**Figure 5C** and **Supplementary Figure 5C**). Moreover, only CD2^+^ TCR-γδ T cells showed an intracellular expression of perforin. In contrast to the CD2^+^ expression, variation within the pigs was smaller in young piglets but increased with age. Within CD2^+^ TCR-γδ T cells, increased frequencies of perforin^+^ cells were detected after the transfer to Vienna and the first vaccinations starting at week 44 (**Figure 5D** and **Supplementary Figure 5D**). The rate of perforin expressing cells showed a high variation but the overall development of increasing expression after the booster immunization was seen in week 80.

### Total number of CD8 T cells and CD27/perforin-defined subsets

**Figure 6:**
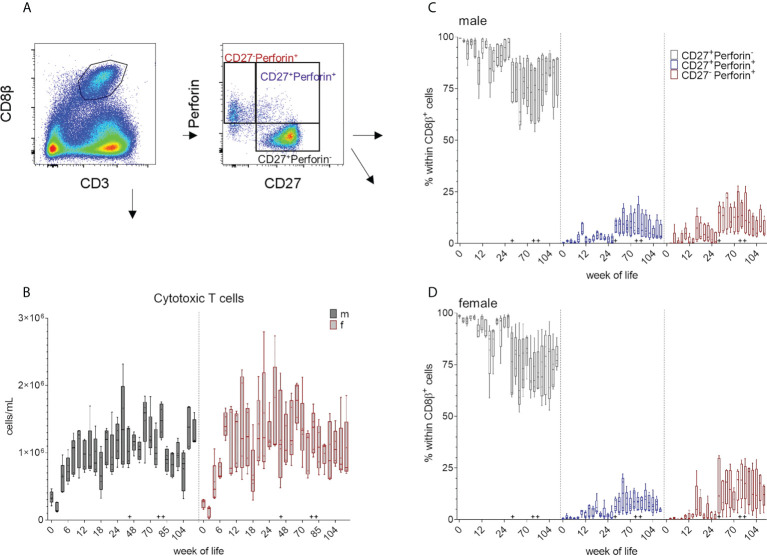
**(A)** Gating strategy to distinguish CD8β^+^ T cells and CD27/perforin-defined subsets on the basis of a representative EGM (male, 18 weeks of age). **(B)** Total numbers of CD3^+^CD8β^+^ T cells were determined as described above over a study period of 126 weeks. Sexes are represented by black (male) and red colors (female). **(C, D)** Development of the relative composition of CD27/perforin-defined phenotypes over a study period of 126 weeks **(C)** males, **(D)** females. + marks the first bleeding time point after transfer and vaccination, ++ marks the first bleeding time point after booster vaccinations.

Porcine MHC-class I restricted cytolytic CD8 T cells (CTLs) can be characterized by their co-expression of CD3 and CD8β^+^ (**Figure 1** and **Figure 6A**). Both sexes showed a decrease in the total cell numbers of CD8^+^ T cells between weeks zero and two, followed by an increase until week ten (males) and week eight (females). An increase around week 60 was followed by a decrease in total cell counts within both groups (**Figure 6B** and supplementary Figure). Overall, no specific trend was observed over the whole study period, but a variation within the median values of CD8^+^ cell frequencies was present. This was independent of sex and the distinct increases in the median CD8^+^ T cells counts (e.g. week 80) likely reflects booster immunizations. As previously described, CD8β^+^ T cells show different phenotypes depending on their CD27 and perforin expression (18, 34, 35) (**Figure 1** and **Figure 6A**). Since CD27 is known to be expressed on naive CD8β^+^ CTLprecursors, CD8β^+^ T cells with down-regulated CD27 expression represent more differentiated CTL phenotypes e.g. CTLeffector cells, while CD8β^+^CD27^+^perforin^+^ seem to represent an intermediate differentiation stage. We postulate, that these two CD8β^+^perforin^+^ subsets show in addition the phenotype of intermediate and terminally differentiated CTLs, respectively (45). In the first weeks of this study, most of the analyzed CD3^+^CD8β^+^ T cells showed a CD27^+^perforin^-^ phenotype (**Figure 6C, D** and **Supplementary Figure 6C, D**). An increase in the more differentiated CD27^+^perforin^+^ and CD27^-^perforin^+^ phenotypes could be detected after week 44 that continued until the end of the study. As expected, this correlated with a relative decrease of the naive CD8β^+^ phenotype. These results indicate that the various vaccinations and the change of surroundings elicited an increase of this more differentiated CD8β^+^ phenotypes.

### Total number of CD4^+^ T cells and their CD8α/CD27^-^defined subsets

**Figure 7:**
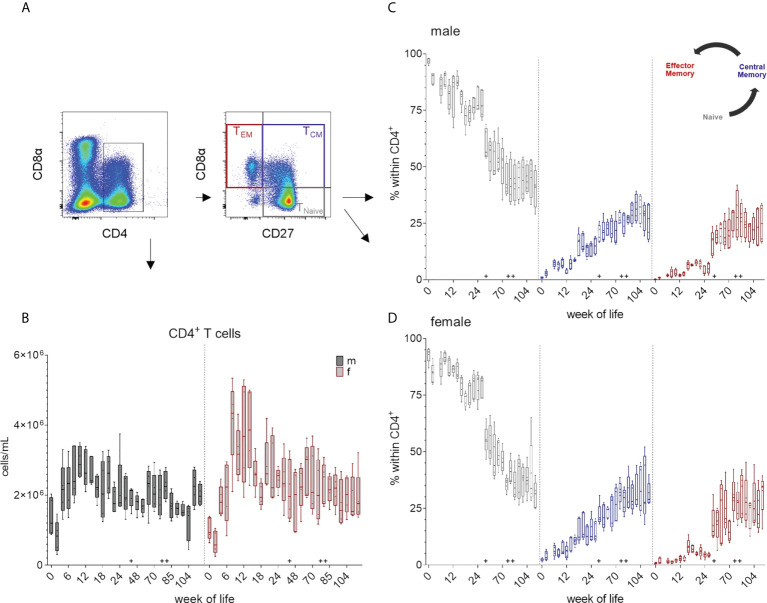
Total numbers of CD4^+^ T cells and the development of T_CM_ and T_EM_ CD4^+^ cells over a study period of 126 weeks. **(A)** Gating strategy to distinguish CD4 T cells and CD8α/CD27-defined subpopulations on the basis of a representative EGM (male, 18 weeks of age). **(B)** Shows the total CD4^+^ T cell counts calculated as described above. Sexes are represented by black (male) and red colors (female). **(C, D)** Changes in the composition of the CD4/CD8α/CD27-defined T cell subsets representing T_Naive_ (CD4^+^CD8α^-^CD27^+^), activated and T_CM_ (CD4^+^CD8α^+^CD27^+^) and T_EM_ (CD4^+^CD8α^+^CD27^-^) in males **(C)** and females **(D)**. + marks the first bleeding time point after transfer and vaccination, ++ marks the first bleeding time point after booster vaccinations.

The expression of CD4 was used as a marker to identify T helper cells. For a further identification of distinct differentiation stages of CD4^+^ T cells, CD8α expression was studied in combination with the expression of CD27. (**Figure 1** and **7A**). As seen for monocytes and CD8β^+^ T cells, total number of CD4^+^ cells initially decreased (weeks zero and two) and a first increase was observed until week ten in males and week eight in females (**Figure 7B** and **Supplementary Figure 7B**). Data of females indicated generally higher median values of CD4^+^ cells between weeks 8 – 14 compared to males. Here, a minor increase in median counts occurred at week 44 compared to week 26, whereas in females there was a decrease in total cell counts. Changes due to vaccination or altered housing conditions did not show a clear trend in total CD4^+^ T cell counts. In contrast, changes in numbers of the respective CD8α/CD27^-^defined CD4^+^ T cell subpopulations were observed over time (**Figure 7C, D** and **Supplementary Figure 7C, D**). Regardless of sex, most CD4^+^ T cells showed a naive CD4^+^CD8α^-^CD27^+^ phenotype at birth and the following weeks. This was followed by a continuous decrease of this naive subset and an increase of the more differentiated phenotypes over time, especially of cells showing an activated and central memory CD4^+^CD8α^+^CD27^+^ phenotype. CD4^+^ T cells with a CD8α^+^CD27^-^ phenotype of effector memory cells first appeared stronger at week 16 and increased after the transfer to Vienna and the vaccination against different pathogens (week 44). At the end of our study, the three CD4/CD8α/CD27^-^defined phenotypes contained nearly equal levels of cells in females, whereas in males' naive CD4^+^ T cells were still slightly higher.

### Antigen-specific immune response

**Figure 8:**
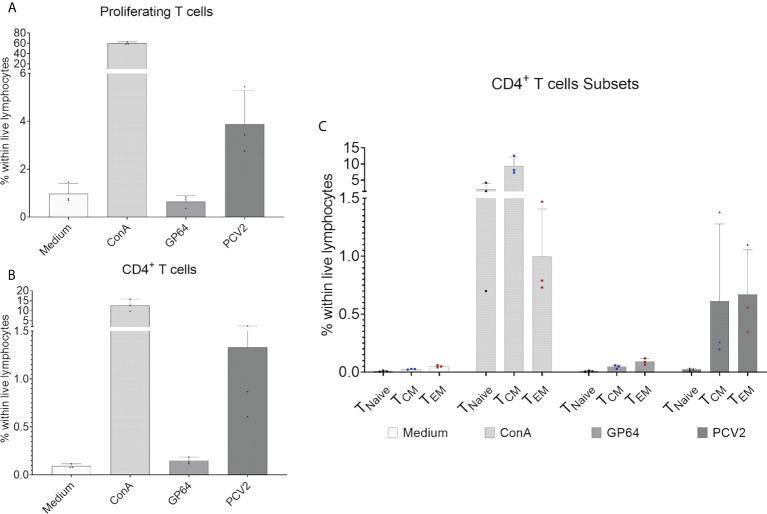
Proliferation assay of CellTrace™Violet-stained PBMCs after restimulation with baculovirus-expressed PCV2-ORF2 protein. PBMCs cultivated in medium or stimulated with an irrelevant baculovirus-expressed protein (GP64) were used as negative controls, ConA-stimulated PBMCs served as positive control. **(A)** Shows the percentage of reacting T cells within live lymphocytes of three representative EGMs. **(B)** shows the percentage of proliferating CD4^+^ T cells within live lymphocytes of three EGMs. **(C)** Shows the reactivity of the CD8α/CD27-defined CD4^+^ T- cell subpopulation of three EGMs. Naive CD4^+^ T cells (T_Naive_), central memory CD4^+^ T cells (T_CM_) and effector memory CD4^+^ T cells (T_EM_).

To investigate the development of an antigen-specific T-cell response after vaccination we focused on CD4^+^ T cells and their proliferative capacity after *in vitro* restimulation with the vaccine antigen the baculovirus-expressed PCV2-ORF2 protein. In addition, we analyzed the production of tumor necrosis factor alpha (TNF-α) and interferon gamma (IFN-γ) as we expected a Th1 response within CD4 cells when using a vaccine against a viral pathogen (46, 47). Three pigs were randomly chosen and an *in vitro* antigen-specific restimulation assay was performed over four days. The vaccine antigen induced a proliferation of total CD3^+^ T cells within the tested pigs (**Figure 8A**), while reactivity against the control antigen GP64 was comparable to the medium control. For CD4^+^ T cells, PCV2-stimulated cells showed a clear PCV2-ORF2-specific proliferative response while the control groups failed to induce a response (**Figure 8B**). For a more detailed analysis of the responding CD4^+^ T cells, we investigated the expression of the differentiation antigens CD8α and CD27. Whereas all CD4^+^ CD8α/CD27^-^defined subpopulations showed a clear reactivity after stimulation with ConA that was used as positive control, only CD4^+^CD8α^+^ T cells with the phenotype of CD27^+^ central and CD27^-^ effector memory cells showed an antigen-specific proliferative reactivity against PCV2-ORF2 (Figure 8C). This confirms T cell memory function of CD8α^+^CD27^+/-^ CD4^+^ T cell subpopulations in EGMs. The gating strategy is shown in **Supplementary Figure 8**.

**Figure 9:**
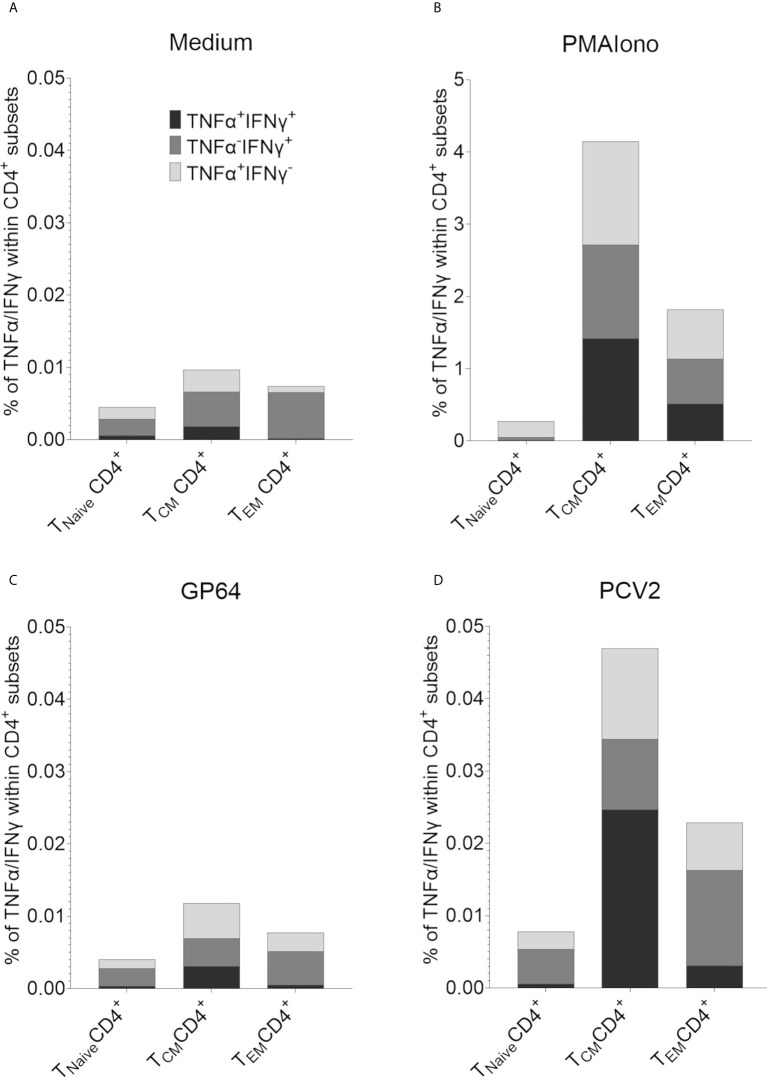
Intracellular cytokine staining for the detection of antigen-specific TNF-α and IFN-γ producing T cells after restimulation with PCV2-ORF2 protein. **(A-D)** Distribution of TNF-α and IFN-γ within T_Naive_, T_CM_ and T_EM_ CD4^+^ cells after 18h of stimulation summarized for six different EGMs.

In addition, an antigen-specific reactivity and recall capacity of these two CD4^+^CD8α^+^ T cell subpopulations could be further characterized by intracellular cytokine staining for TNF-α and IFN-γ. Here, PBMCs of six randomly chosen pigs of our herd were restimulated with PCV2-ORF2 overnight. A control staining without TNF-α and IFN-γ can be found in **Supplementary Figure 9**. CD4^+^ T cells cultivated in medium showed hardly any cytokine production of TNF-α and IFN-γ with less than 0.01% for all CD8α/CD27^-^defined T cell subsets (**Figure 9A**). Stimulation with PMA/Ionomycin as positive control increased the percentage of cytokine-producing CD4^+^ within the CD8α^+^CD27^+^ TCM and the CD8α^+^CD27^-^ TEM with nearly equal distribution of TNF-α^+^IFN^-^γ^-^, TNF-α^-^IFN-γ^+^, and double positive TNF-α^+^IFN-γ^+^ producing cells (**Figure 9B**). Stimulation with control antigen GP64 showed only marginal cytokine production, comparable to the medium control (**Figure 9C**). In contrast, restimulation with PCV2-ORF2 resulted in an increase of all three TNF-α/IFN-γ-defined subsets in both CD8α^+^ TCM and TEM. Of note, TNF-α^+^IFN-γ^+^ double positive cells dominated within the TCM subset, while TNF-α^-^IFN-γ^+^ cells dominated in the TEM subset. As expected, the Tnaive subsets showed a response comparable to the unstimulated groups (**Figure 9D**).

## Discussion

To increase the knowledge on the immune system of EGMs we investigated the postnatal immune system development and further changes within the composition of immune cell populations after vaccination and transfer to different housing conditions, thus exploring the memory development of TCR-αβ T cells. Furthermore, we scrutinized the antigen-specific immune response *via* cytokine production and proliferation capacity of CD4^+^ T cells by using PCV2-ORF2 antigen for restimulation.

The overall trend of increasing numbers within leukocytes and lymphocytes during early postnatal development fits previous findings in pigs (18), although we could find higher levels of total cell counts of both leukocytes and lymphocytes over the first six months in EGMs. In comparison with multiparous large white sows aged 33.5 ± 9.6 months that had 12.1 × 10^6^/mL ± 2.1 × 10^6^/mL (±SD) (48), we found slightly fewer leukocytes (mean value of 9.5 × 10^6^/mL ± 2.8 × 10^6^/mL) within the EGM population at the end of our study. Nevertheless, the percentage of lymphocytes was higher in EGMs (59.9% ± 5.9% lymphocytes within leukocytes) versus 44.7% ± 10.2% in Large White breed (48). We have to mention that effects of pregnancy to the immune system are not taken into consideration here, only age in contrast to the mentioned study. Using another data set for comparison (49), we find comparable means around one year but slightly lower leukocyte numbers in the younger EGMs. For 3.5 – 4 months (49), an average value of 26.9 × 10^6^/mL was found in Duroc - Jersey Swine, while EMGs in our study had a mean of 15.7 × 10^6^/mL ± 2.7 × 10^6^/mL (males) and 14.6 × 10^6^/mL ± 2.7 × 10^6^/mL (females) at 16 weeks of age. At one year of age, the average for males was 13.3 × 10^6^/mL and 16.4 × 10^6^/mL in females in Duroc - Jersey Swine, while EGMs had 15.6 × 10^6^/mL ± 5.4 × 10^6^/mL (males) and 17.0 × 10^6^/mL ± 4.6× 10^6^/mL (females) leukocytes at 60 weeks of age. A similar picture within the two breeds was found looking at the percentage of lymphocytes around 4 months of age: Duroc - Jersey Swine had an average of 63% of lymphocytes while EGMs had an average of 60.6% ± 10.7%. EGMs showed slightly higher levels when we compared ≥ 1 year old pigs. Duroc - Jersey Swine had 55% (males) and 54% (females) whereas EGMs had 65% ± 8% (males) and 68% ± 6% (females) of lymphocytes on average. Apart from this, our findings generally confirm the findings cited by Weiss and Wardrop that age related changes in EGMs are similar to domestic swine (49). As EGMs are important as models in translational research and experimental medicine, comparison to NHP and primates is of importance. Compared to other species, the levels of leukocytes fit the ranges found, for example, in adult Macaca fascicularis and Macaca mulatta aged between 48 – 96 months. Here, leukocyte counts of 7.5 × 10^6^/mL ± 2.1 × 10^6^/mL (males) and 7.9 × 10^6^/mL ± 2.7 × 10^6^/mL (females) were found and lymphocytes with an average of 36.7% ± 17.5% (males) and 39.6% ± 13.1% (females) in Macaca (50). In adult humans, the reference values are 4 – 10 × 10^6^/mL leukocytes and 25 – 40% of lymphocytes according to Pschyrembel, 2020 (51). Linking it to week 126 in our study, we find a mean of 10.1 × 10^6^/mL ± 3.4 × 10^6^/mL (males) and 8.8 × 10^6^/mL ± 2.4 × 10^6^/mL (females) of leukocytes which fits human reference values well, whereas we found a higher number of lymphocytes in EGMs: 58.9% ± 2.1% (males) and 60.7% ± 7.9% (females). When comparing EGMs to other species one has to consider age as our pigs at the age of week 126 relate more to young adult humans. Investigating total monocyte numbers and reference values in humans (51) 3 – 7% were found within leukocytes in adult humans whereas EGMs showed a mean of 19.4% ± 3.8% at week 126. Monocyte frequencies are also higher compared to NHP (50) and to > 1year Duroc - Jersey Swine with an average of 5-8% within leukocytes (49).

In general, EGMs showed comparable numbers of B cells to domestic swine (18) over the first six months of life although their immune system had not been challenged by vaccination yet. Pschyrembel, 2020 (51) lists 3 – 14% as reference values for adult humans while we found 14.8% ± 6% in EGMs at week 126. Using reference data by Caldwell et al. (52) percentages above 20 can be found in Cynomolgus from Vietnam, China and the Philippines whereas the ones from Mauritius are below 20%. This underlines a variety between and within species but also potential similarities. Here it would be of interest to gain insight into older EGM to see how their B-cell development progresses, additionally a more detailed characterization of B-cell subpopulations would be of interest.

The postnatal development of NK cells within EGMs followed a comparable trend as for domestic swine over the first months of life (18). In relation to human NK cell proportions with 2 – 18% (53), EGMs in our study showed NK cells levels at the lower end of the reference values at week 126 (3,4% ± 1,6%). This is potentially related to age, as another study showed an increase in NK cells with age in humans (54). Here, further research will be necessary to investigate older pigs as well. Our study also found a high variation in NK-cell proportion within males and females. As NK cells play a potential role in rejection of xenotransplants between pig-to-human (55) it is important to investigate similarities and differences between the species. One major difference between humans and pigs seems to be the expression of NKp46. NKp46 has been reported to be conserved in mammalian species (56) and is expressed by human NK cells independently of their activation status (57). In contrast, recent research in domestic swine has shown that NKp46 is not expressed by all porcine NK cells (26) which is also true in EGMs. Previous work showed that NK cells with the CD8α^dim/+^NKp46^high^ phenotype are in an activated state (27). Similar to domestic swine (18) we encountered a diverse NKp46 expression within EGMs, which continued to be present over the whole study. This potential age-dependent decrease of NKp46^high^ cells agrees with findings in humans that show a decrease of the expression of the receptor NKp46 in elderly individuals (58).

Swine are known to have a high abundance of TCR-γδ T cells (28, 29, 59), which is also true for EGMs according to our data. However, within our study we did not find an total increase around month six as shown in an earlier study for domestic swine (18). TCR-γδ T cells are now known to bridge innate and adaptive immunity with a wide variety of functions such as killing of infected cells, immune-regulation, or potentially even formation of memory cells (32, 60). In humans, TCR-γδ T cells represent only a small proportion of T cells with around 1 – 10% within PBMCs (61) and, in contrast to TCR-αβ T cells, they can be activated in an MHC-independent manner (62). The role of TCR-γδ T cells in the immune response is still under ongoing investigation and they are suspected to play a role in cancer, having both protective potential but also playing a possible role in tumor growth depending on their phenotype (63). As the frequency of TCR-γδ T cells in swine is much higher, pigs provide a better platform to investigate those cells compared to NHPs or humans. Furthermore, in swine, two distinct TCR-γδ cell populations are present: CD2^+^ and CD2^-^. It has been reported in the past that TCR-γδhigh expression correlates with the CD2^-^ phenotype whereas TCR-γδ^medium^ expression associates with CD2 expression (44). One major difference within the two subsets is that CD2^-^ TCR-γδ T cells do not exist in humans to our current knowledge. Recent findings therefore supported the idea that CD2 expression might define two specific lineages and therefore CD2^-^ T cells cannot gain CD2 (44). Lately this dogma has been questioned as CD2 expression could be induced after *in vitro* stimulation in CD2^-^ TCR-γδ T cells (32). Furthermore, CD2^-^ TCR-γδ T cells seem unresponsive to antigenic stimuli, whereas CD2^+^ TCR-γδ T cells show similarities to human TCR-γδ cells in their reactivity (22). Additional research addressing similarities and differences within human and swine would be of interest in the future to establish a potential model for enhanced TCR-γδ T-cell research. Here EGMs offer much potential. Increased presence of TCR-γδ T cells offers a good possibility to study this subset in a pig model as these cells gain increasing recognition in disease studies (64–67), but reduced presence of this cell population in humans must be taken into account.

Total numbers of CD8β^+^ T cells of our current study match previous findings during the first six months in domestic swine (18). Numbers of CD8 T cells in humans were between by 21 – 36% (51) in comparison to 19,9% ± 2,9% at week 126 in our study. Cynomolgus monkeys show a mean frequency closer to EGMs between 22 – 26% (52). We did not encounter an increase of total counts of CD8β^+^ T cells after transfer and the first vaccinations but did see changes in the CD27/perforin-defined phenotypes of the investigated cells. During the early postnatal development, most of the CD8β^+^ T cells represented the naive CD27^+^ phenotype. As seen in domestic swine (18), the increase of perforin over time occurred in relation to an overall decrease of CD27. A first minor increase arose at week 4, which marks the time of weaning and therefore might be related to it. After the transfer of the animals, vaccination, and the change in housing conditions an increased frequency of perforin^+^ cells appeared within the two phenotypes CD27^+^perforin^+^ and CD27^-^perforin^+^. Those findings in the study emphasize the idea that the perforin upregulation and CD27 downregulation is related to memory development. This was verified by Lagumdzic et al. (45) studying marker combinations of CD27 and CD11a to define differentiation phenotypes and use these FCM-sorted subsets for further analyses with next generation RNA sequencing. The study showed that after stimulation with PMA/Ionomycin PRF1 (Perforin) was found to be upregulated in differentiated CD8 cells but not in naive which relates to our findings (45). Further, the CD27 expression pattern corresponds to current knowledge with human CD8 T cells. In a review by Martin et al. (68) Tem cells were defined as CD27^-^ whereas Tcm cells were still CD27^+^. Here, recent findings by Lagumdzic et al. in domestic swine showed high similarities within the effector subpopulations compared to human and mice. For example, TBX21, BLIMP1, ID2, and STAT4 were found to be upregulated in terminally differentiated porcine CD8^+^ T cells. Furthermore, human and swine showed higher orthologue gene overlaps than human and mice, which underlines the potential of swine as fitting animal model in translational research (45). Due to the breeding background of EGMs and the findings in our study similar findings in EGM can be expected.

In humans and NHP, expression of CD8α within CD4^+^ T cells has been an ongoing research focus (69, 70). In swine, CD4 T cells expressing CD8α were described decades ago as hallmark of antigen-experienced cells (21, 23, 71) and are in the meantime well characterized (22, 35, 40, 42). As mentioned above, CD4^+^CD8α^-^CD27^+^ are considered as naive, CD4^+^CD8α^+^CD27^+^ phenotypes belong either to activated cells or central memory (TCM) cells and CD4^+^CD8α^+^CD27^-^ are defined as effector memory (TEM) T cells. As expected, the CD4^+^CD8α^-^CD27^+^ phenotype was the most frequent during the early postnatal development in EGMs. Over time, an increase in the CD4^+^CD8α^+^CD27^+^ and CD4^+^CD8α^+^CD27^-^ subsets occurred after transfer to Vienna and vaccination. This underlines the assumption that naive, central and effector memory phenotypes are also true for EGMs. Looking at the frequency of CD4^+^ T cells in different species we find comparable numbers that differ more in humans and pigs within one species compared to NHPs. Within humans, 32 – 50% CD4^+^ T cells are the relative numbers (51) in comparison to different Cynomolgus strains showing 29 – 33.5% (52), while our findings at week 126 were 35.1% ± 5.1%) for EGMs. In humans, naive CD4^+^ cells normally are described as CD45RO^-^CCR7^+^CD28^+^ while TCM show CD45RO^+^CCR7^+^CD28^+^ and TEM are CD45RO^-^CCR7^-^CD28^-^ (22). Recently, the expression of CD28 in swine was investigated (72) and reported naive CD4^+^CD8α^-^CD27^+^CD28^+^, central memory with CD4^+^CD8α^+^CD27^+^CD28^+^ phenotype and effector memory showing either CD4^+^CD8α^+^CD27^-^CD28^+^ or CD4^+^CD8α^+^CD27^-^CD28^low/-^ expression.

As previously described, an *in vitro* restimulation of PBMCs derived from PCV2-vaccinated pigs with PCV2-ORF2 induces IFN-γ and TNF-α production in domestic swine (46). We were able to show that this is also true for EGMs. As excepted, naive CD4^+^ cells scarcely produced TNF-α and IFN-γ after in vitro restimulation while we found PCV2 antigen-specific TNF-α^+^ and/or IFN-γ^+^ producing cells within CD27^+^ central and CD27^-^ effector memory CD4^+^CD8α^+^ T cells. In contrast to the findings by Koinig et al. (46), we did not find an even distribution within central and effector memory cells. In our experiments, central memory CD4^+^ T cells seemed to be the main producers of TNF-α and IFN-γ. This was surprising as it is common knowledge for the human recall response that not central but effector memory CD4 T cells are the main cytokine producing cells after restimulation (73, 74). This could be due to the *in vitro* setting for the restimulation. In addition, our proliferation results confirmed the antigen-specific immune response capacity of the PBMCs *in vitro*, especially as we only found TCM and TEM responding within the proliferating CD4^+^ T cells. The ability to examine this antigen-specific immune response of vaccinated pigs *in vitro* are first steps to an efficacious cell culture system that can be used to investigate immune modulating agents and compare their effects in swine to humans. An antigen-specific immune response always has the advantage of representing a situation more closely related to a real-life situation compared to using for example T cell mitogens or superantigens.

In summary, in our study we investigated the postnatal development of EGMs to gain knowledge about usability of reagents and mAbs created for domestic swine, the postnatal immune system development under conditions close to SPF, and adaptations of the immune system and memory development after changed housing conditions and vaccination. We were able to confirm that all reagents and mAbs created for domestic swine are usable and we were able to follow a development of certain immune cell subsets over time in the EGM model. Within the CD4^+^ T cells, we were able to track the development of memory subsets over time, which is of high interest in vaccination studies. The work focusing on restimulating capacities after vaccination is of value as it allows to gain insight on vaccine success and enables an *in vitro* approach for testing highly specific immune modulating drugs as mentioned above.

## Data availability statement

The raw data supporting the conclusions of this article will be made available by the authors, without undue reservation.

## Ethics statement

The animal study was reviewed and approved by in Denmark by the Danish authorities (license 2019-15-0201-01622) and in Austria by the institutional ethics committee, the Advisory Committee for Animal Experiments (§12 Animal Experiments Act - TVG), and Austrian Federal Ministry of Education, of Science and Research (reference BMBWF-68.205/0198-V/3b/2019).

## Author contributions

Concept development: CPSP, SJ, MWS, KHM and AS. Experimental work: CPSP, MS. Caretaking of the pigs and bleeding: AL, CK, SD, HK and VM. Data analysis: CPSP, AS. Data Interpretation: CPSP, KM, EL and AS. Figure Preparation: CPSP. Writing: CPSP, AS and SS. All authors read and approved the final manuscript.

## Funding

The authors declare that this study received funding from Merck HealthCare KGaA. The funder was not involved in the study design, collection, analysis, interpretation of data, the writing of this article, or the decision to submit it for publication.

## Acknowledgments

We thank Wilhelm Gerner for helping in developing the panels for FCM analysis. We also thank the team of Ellegaard Göttingen Minipigs A/S for taking care of and bleeding the pigs until six months of age.

## Conflict of interest

Authors SJ and MS were employed by company Merck KGaA.

The remaining authors declare that the research was conducted in the absence of any commercial or financial relationships that could be construed as a potential conflict of interest.

## Publisher’s note

All claims expressed in this article are solely those of the authors and do not necessarily represent those of their affiliated organizations, or those of the publisher, the editors and the reviewers. Any product that may be evaluated in this article, or claim that may be made by its manufacturer, is not guaranteed or endorsed by the publisher.
