# Supplementary material for: Characterization of the immune system of Ellegaard Göttingen Minipigs - An important large animal model in experimental medicine
Source: Front Immunol. 2022 Sep 20;13:1003986. doi: 10.3389/fimmu.2022.1003986 (PMC9531550; doi:10.3389/fimmu.2022.1003986)
Supplement: Supplementary file 1 [file DataSheet_1.docx]

Supplementary Material

##
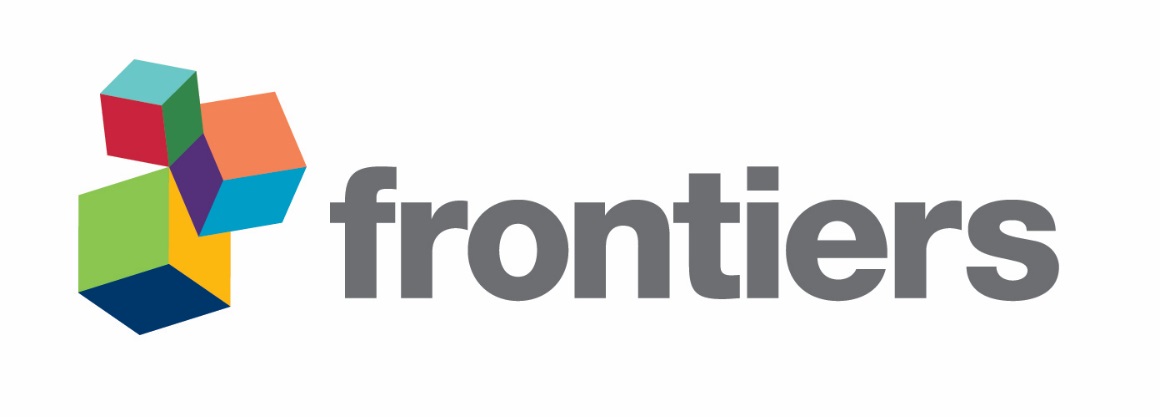


## Supplementary Table

**Table 1**

Comparison of immune cells of domestic swine, EGMs and humans.

| **Cell population** | **Species** | Large White breed ^48^ | EGMs | Humans^51^ |
| --- | --- | --- | --- | --- |
| Leukocytes/ml |  | 12.1 ± 2.1 × 10^6^/ml | 9.5 ± 2.8 × 10^6^/mL | 4-10 × 10^6^/mL |
| Lymphocytes ^% within Leukocytes^ |  | 44.7 ± 10.2% | 59.9 ± 5.9% | 25-40 % |
| Monocytes |  | 4.5 ± 1.4% | 19.4 ± 3.8% | 3-7 % |
| NK cells |  | 2,6 ± 2,7% | 3,4 ± 1,6% | 2-18% ^53^ |
| B cells |  | 6,0 ± 2,5% | 14.8 ± 6,0% | 3-14% |
| CD4 T cells |  | 36,3 ± 4,6% | 35.1 ± 5.1% | 32-50% |
| CD8 T cells |  | 37,3 ± 7,0% | 19,9 ± 2,9% | 21-36% |
| TCR-γδ T cells |  | 19,1 ± 7,8% | 14,7 ± 2,6% | 1-10% ^61^ |

## Supplementary Figures

Supplementary Figure 1


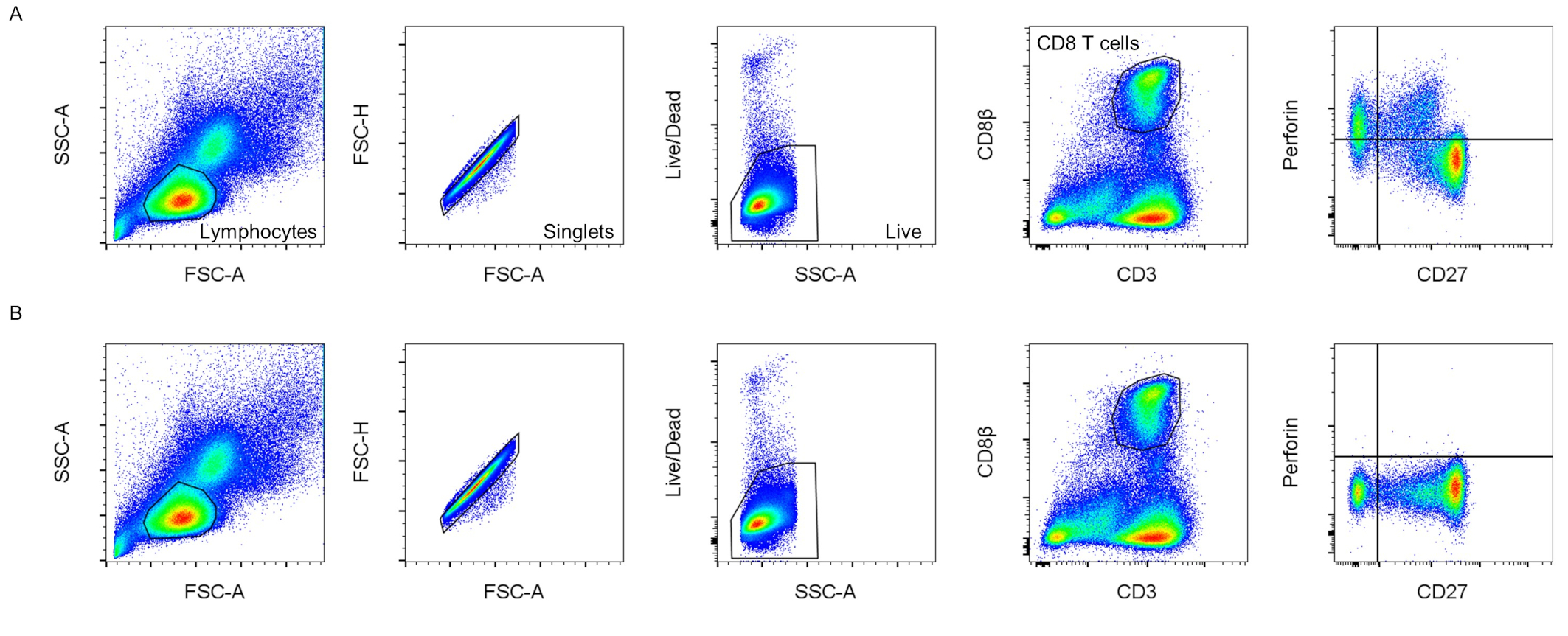


**Supplementary Figure 1:** FMO control staining of the intracellular marker Perforin. (A) shows the sample including all markers whereas (B) is the according control without the mAb against Perforin. A representative male animal aged 126 weeks was chosen.

Supplementary Figure 2:


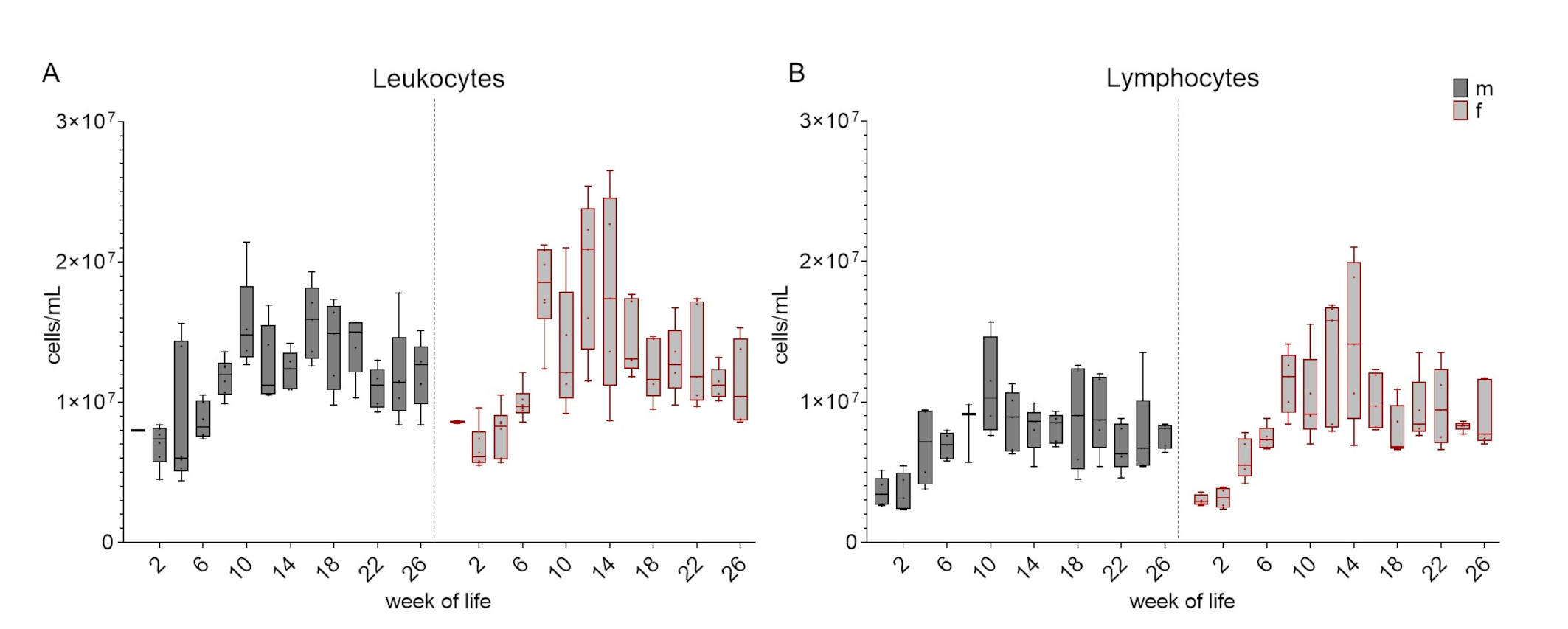


**Supplementary Figure 2:** Corresponding to Figure 2, showing the first 26 weeks of the study.

Supplementary Figure 3

.**Supplementary Figure 3:** Corresponding to Figure 3, showing the first 26 weeks of the study.


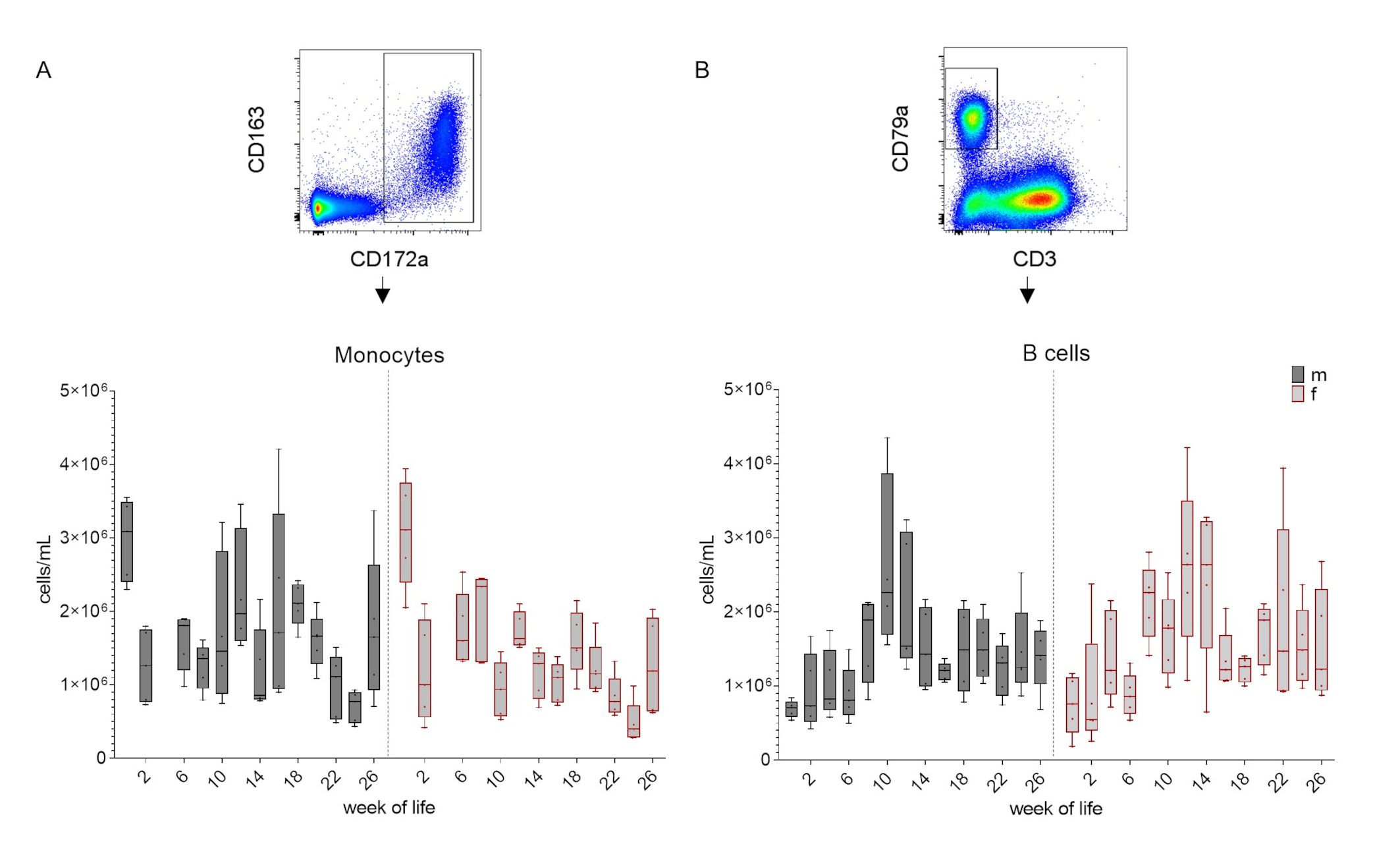


Supplementary Figure 4

**Supplementary Figure 4:** Corresponding to Figure 4, showing the first 26 weeks of the study.


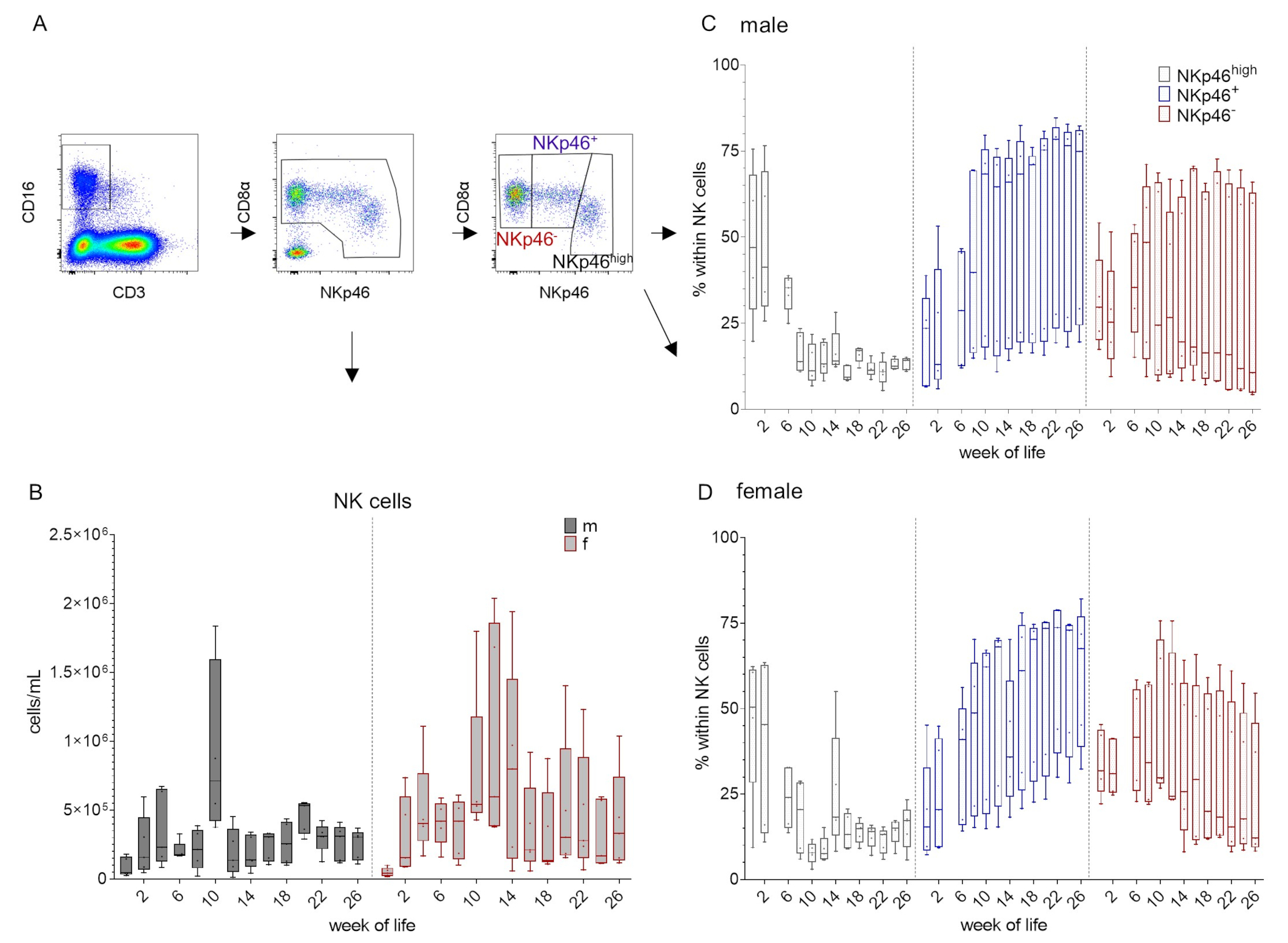


Supplementary Figure 5

**Supplementary Figure 5:** Corresponding to Figure 5, showing the first 26 weeks of the study.


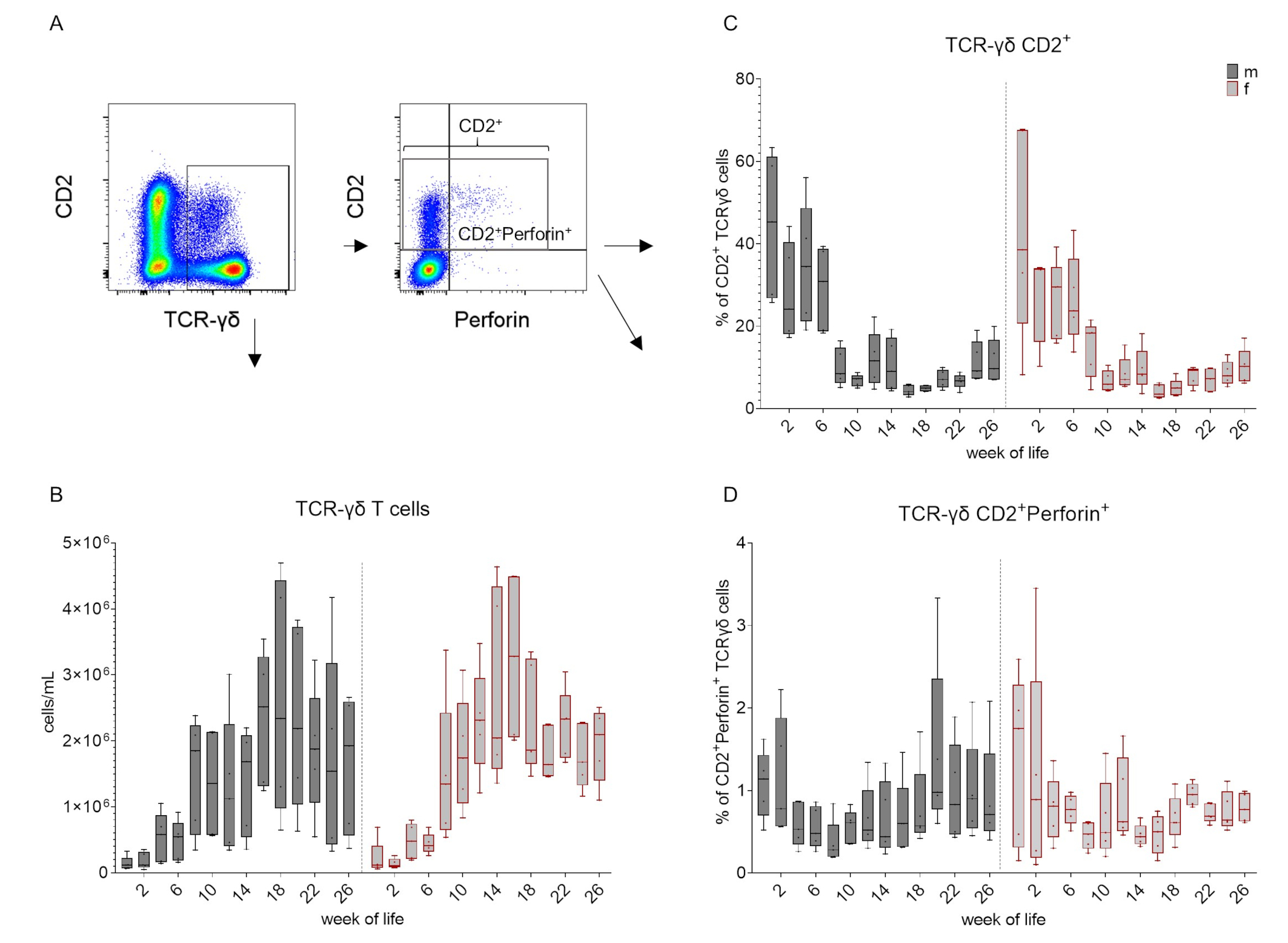


Supplementary Figure 6


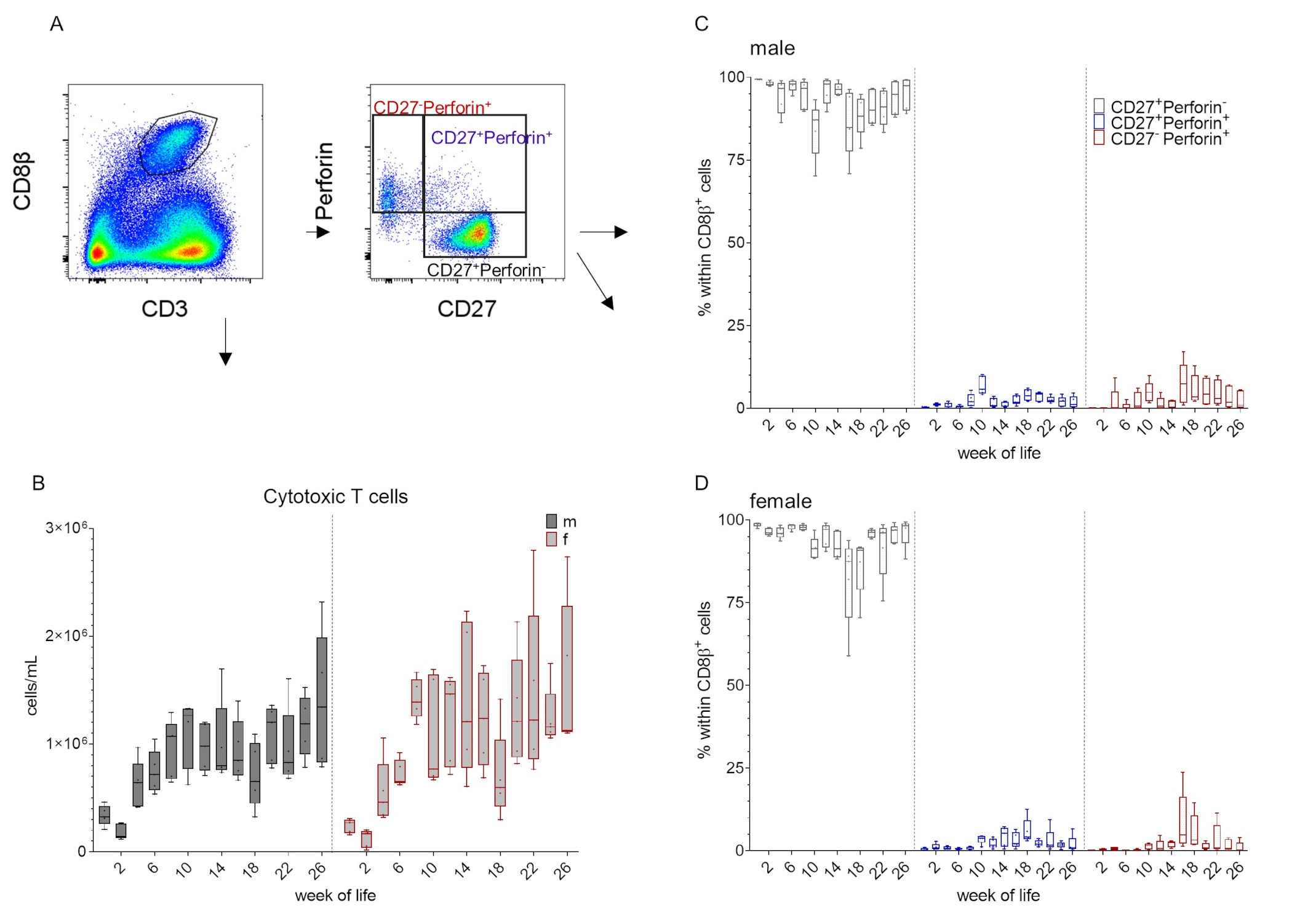


**Supplementary Figure 6:** Corresponding to Figure 6, showing the first 26 weeks of the study.

Supplementary Figure 7


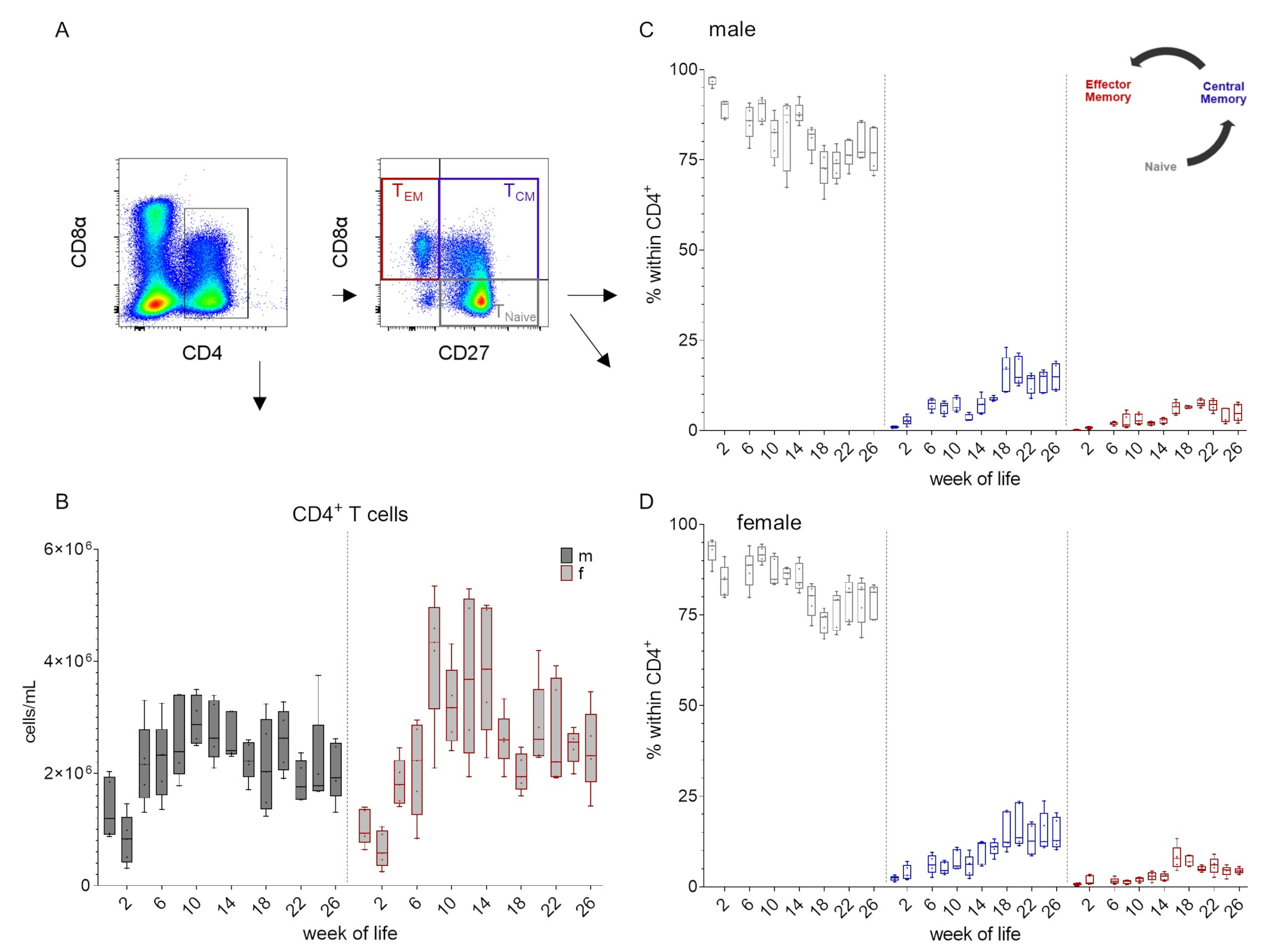


**Supplementary Figure 7:** Corresponding to Figure 7, showing the first 26 weeks of the study.

Supplementary Figure 8


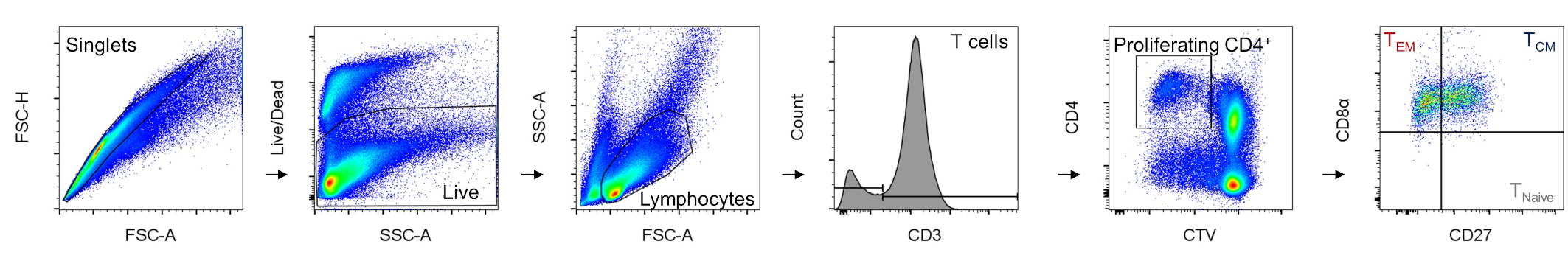


**Supplementary Figure 8:** Gating strategy for the analysis of proliferating CD4^+^ T cells and the respective CD8α- and CD27-defined subpopulations. A representative animal after restimulation with PCV2 was chosen for the gating strategy.

Supplementary Figure 9


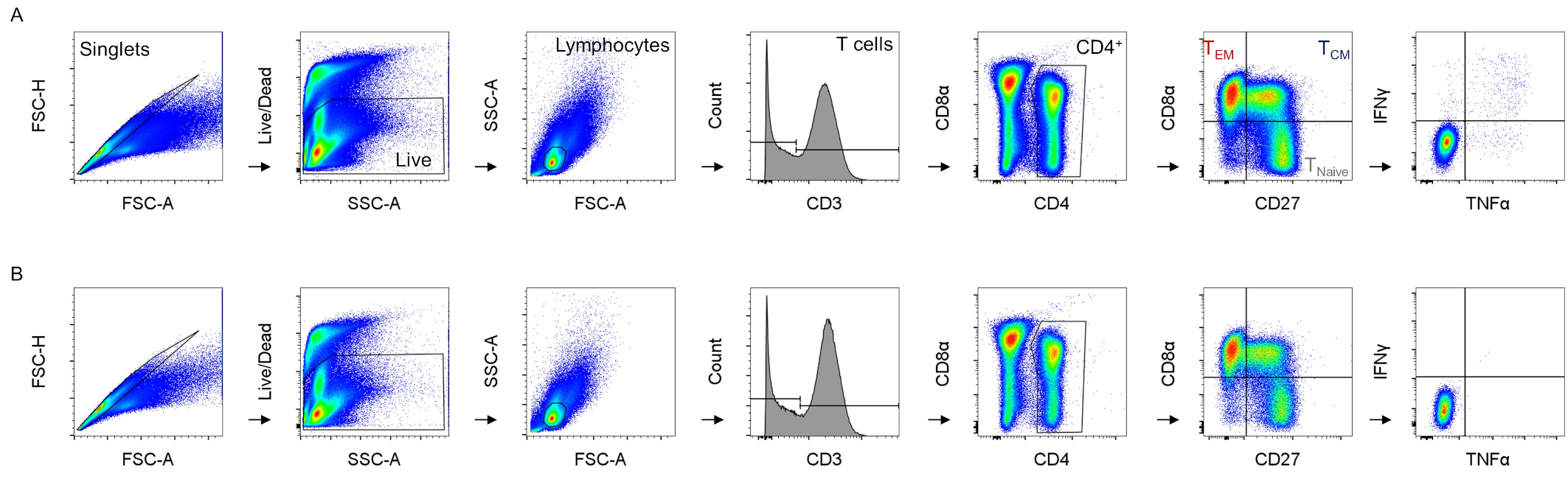


**Supplementary Figure 9:** Gating strategy for ICS after PCV2-ORF2 restimulation. Gating strategy for the identification of cytokine producing T cell subpopulations of a representative EGM after a restimulation with PCV2-ORF2. Within CD3^+^ T cells CD4^+^ T cells, were further analyzed regarding to their CD8α and CD27 expression and the production of TNF-α and IFN-γ within the three CD4^+^ subsets - naïve, central and effector memory were analyzed to identify cytokine producing CD4 T cell subsets. (A) shows the samples including all markers whereas (B) is the according control. Here both cytokines were not stained with the specific mAbs.
